# Breast cancer immunogenicity as a spectrum: evolving concepts and therapeutic implications

**DOI:** 10.1172/JCI207634

**Published:** 2026-10-01

**Authors:** Jasmine Kay, Julia R. Dixon-Douglas, Michael A. Harris, Courtney T. van Geelen, Sherene Loi

**Affiliations:** 1 The Peter MacCallum Cancer Institute, Melbourne, Victoria, Australia.; 2 The Peter MacCallum Department of Oncology, University of Melbourne, Melbourne, Victoria, Australia.

## Abstract

Immunotherapy has revolutionized the therapeutic landscape for many cancers, but its application in solid tumors has lagged. There is now evidence that immunotherapy can improve outcomes in triple-negative breast cancer, but hormone receptor–positive (HR^+^) breast cancer has traditionally been considered immunologically cold. However, emerging evidence challenges this binary paradigm, suggesting that a biologically relevant subset of HR^+^/human epidermal growth factor receptor 2–negative (HER2^–^) tumors exhibit meaningful immunogenic features and clinically relevant sensitivity to immune-based treatment. In this Review we summarize the current understanding of immunogenicity and clinical use of immune-based treatments across breast cancer subtypes. We argue for a broader view of a spectrum of breast cancer immunogenicity and highlight the importance of host factors, including parity and lactation history, in shaping antitumor immunity. Improved identification of immunologically active subsets and deeper mechanistic insight will be essential to expand the therapeutic benefit of immunotherapy to broader patient cohorts and to refine care of patients with breast cancer.

## Introduction

Breast cancer remains the leading cause of cancer-related mortality in women, and the incidence is continuing to rise (1–4). Breast cancer is a highly heterogenous disease, which has typically been subdivided based on the expression or absence of hormone receptors (HRs) and human epidermal growth factor receptor 2 (HER2) by immunohistochemistry (IHC). HR^+^ breast cancers (HR^+^ BCs) are defined by positive estrogen receptor (ER) and/or progesterone receptor (PR) expression. Positive HER2 expression denotes the HER2^+^ breast cancer (HER2^+^ BC) subtype, and triple-negative breast cancer (TNBC) is categorized by the absence of ER, PR, and HER2 expression.

Accounting for roughly 10%–15% of breast cancers (5), TNBC is particularly aggressive, with high risk of distant recurrence after early-stage disease and short survival rates in the case of recurrence, in part due to lack of targeted treatment options (6). Historically, the treatment of TNBC has relied primarily on surgery, followed by adjuvant chemotherapy and radiotherapy, with up to 30% of patients recurring or progressing to metastatic disease (7).

Early-stage HR^+^/HER2^–^ BC accounts for over 70% of new diagnoses and generally has had relatively good prognosis although the persistent risk of late recurrence results in substantial long-term mortality (8). Treatment typically consists of surgery followed by adjuvant radiotherapy and endocrine therapy, with or without chemotherapy (6). However, outcomes remain varied, with some tumors exhibiting aggressive biology (high grade, low ER expression, high proliferative index and genomic scores), associated with higher risk of recurrence, especially among younger patients.

The immune microenvironment of the breast is complex and highly relevant in the context of cancer (9). Indeed, tumor immunity is now acknowledged as a key hallmark of cancer (10), and immunotherapy has become a pillar of cancer treatment (11). Recent advances in immunotherapy, primarily with antibodies against programmed cell death protein 1 and its ligand (anti–PD-[L]1), combined with chemotherapy have markedly improved the response rates and overall survival of patients with TNBC, both in the early and in the metastatic setting (7, 12–14).

Local immunity and its importance within breast tumors have a long history, with early observational studies dating to the 1920s (15). Progress in this field has been spearheaded by an increased understanding of the role of tumor-infiltrating lymphocytes (TILs) and other immune cells within the breast microenvironment. TILs and other infiltrating immune cells have diverse phenotypes that can dictate whether a tumor microenvironment (TME) is considered immune “hot” or “cold,” pro- or antiinflammatory (9, 16). For instance, CD8^+^ TILs have repeatedly been correlated with a “hotter” TME (9) and have long been associated with improved outcomes and recurrence-free survival in breast cancer, dating back to even before receptor testing by IHC was widespread in clinical practice (17). Early studies established a connection between more aggressive subtypes (e.g., higher grade, lymph node infiltrating) and higher numbers of TILs (17). Studies have consistently corroborated the positive correlation between TILs and improved response to treatment (18, 19) and overall patient outcomes (20–24). TILs have also been shown to be prognostic and predictive in HER2^+^ BC, with higher levels of TILs predicting the likelihood of pathological complete response (pCR) to anti-HER2 therapy in combination with chemotherapy (25).

TNBC is considered the most immunogenic breast cancer subtype, with higher levels of TILs relative to other subtypes and a strong correlation between high TILs and favorable long-term outcomes to chemotherapy, particularly in early-stage disease (24). As a result, TNBC has emerged as the model for immunogenicity in breast cancer and is currently the principal indication for immunotherapy in breast cancer. In contrast, HR^+^/HER2^–^ disease has been broadly considered nonimmunogenic, with a “cold” immune microenvironment characterized by a lower level of TILs compared with TNBC or even HER2^+^ disease (23, 26). In HR^+^ disease, TILs have been reported to either have no correlation with outcome (20, 23), or paradoxically have a negative correlation, either as a prognostic (21) or as a predictive biomarker (27). However, recent clinical data have shown that the addition of immunotherapy to neoadjuvant chemotherapy boosts response rates compared with chemotherapy alone in a selected cohort of high-risk, early HR^+^/HER2^–^ BC (28, 29). This indicates that rewiring the immune microenvironment may have a therapeutic role in this subtype and brings renewed focus to characterizing the heterogeneity of the immune microenvironment of HR^+^/HER2^–^ BC to identify patients likely to benefit from immunotherapeutic interventions.

In this Review, we summarize the current concepts of immunogenicity in breast cancer, review established and emerging clinical indications for immunotherapy, and discuss how our understanding of immunogenicity in breast cancer is evolving, specifically in the context of HR^+^/HER2^–^ disease.

## TNBC as the model for breast cancer immunotherapy

Given the prognostic significance of immune infiltration in TNBC, coupled with the success of anti–PD-(L)1 immune checkpoint blockade (ICB) in other immunogenic malignancies, TNBC was an attractive option for ICB trials in breast cancer.

Initial studies in previously treated advanced and metastatic TNBC showed only modest response rates to anti–PD-1 monotherapy with pembrolizumab (objective response rate [ORR] 5%–6% in KEYNOTE-086) (30, 31) and no significant improvement compared to chemotherapy in KEYNOTE-119 (32). Despite these modest results, the long duration of response to treatment and longer overall survival in patients with tumors that were PD-L1 positive by IHC indicated that a subset of patients with TNBC may derive benefit from immunotherapy. The KEYNOTE-355 randomized, phase III study reported a statistically significant improvement in overall survival with the addition of pembrolizumab to first-line chemotherapy in metastatic, PD-L1–positive TNBC, leading to regulatory approvals for pembrolizumab for this indication (12). The anti–PD-L1 antibody atezolizumab is also approved in some regions for TNBC, based on similar results from the IMpassion130 trial (33). Other clinical trials in the metastatic setting have likewise observed some efficacy signals in PD-L1–positive cohorts. However, the overall response rates in the advanced setting remain minimal, and side effects remain a major challenge (Table 1).

Following these landmark trials, the combination of pembrolizumab and chemotherapy has been implemented in early-stage TNBC as well, with improved pCR rates, recurrence-free survival, and overall survival in the neoadjuvant setting. The KEYNOTE-522 trial was the first randomized, phase III trial to show a significant improvements in pCR event-free survival, and overall survival at the cost of increased treatment related toxicity in early-stage TNBC with a 5% increase in grade 3 treatment-related events (73% vs. 78%) (34). Importantly, these results were observed independent of the PD-L1 status of the patients. These results have since been reproduced in other similarly designed neoadjuvant clinical trials, establishing ICB as the standard of care for TNBC (Table 2). Improving the response rates further will require better understanding of biomarker inconsistencies across different trials and efforts to standardize assays in future applications (35).

## Immunogenicity in HER2^+^ BC

Although high levels of TILs have been associated with improved response rates and favorable long-term outcomes in HER2^+^ BC (20, 36, 37), ICB is not currently part of the standard of care, likely reflecting the incredible efficacy of current and emerging HER2-targeted therapies (38). Treatment of HER2+ BC relies on HER2-directed therapies, including trastuzumab, often combined with pertuzumab and chemotherapy, HER2-directed tyrosine kinase inhibitors, and antibody-drug conjugates (ADCs) such as T-DM1 and T-DXd. Notably, many of these approaches have been shown to have immune-mediated mechanisms that contribute to their efficacy (39).

Several clinical trials have shown proof of concept that ICB can have activity in HER2^+^ BC (40–42). However, the addition of atezolizumab to neoadjuvant chemotherapy and dual HER2-targeted blockade did not significantly improve pCR rates in the IMpassion050 clinical trial (43). The reasons for this remain unclear. One possibility is that trastuzumab and pertuzumab already provide substantial priming (25, 44), limiting the potential benefit of the addition of atezolizumab. It is also possible that combining ICB with intense myelosuppressive combination chemotherapy increased toxicity and discontinuation rates and reduced the capacity to generate an effective immune response. As such, while the data suggest immunity is an important aspect of treatment response in HER2^+^ BC, more research is needed to identify the patient cohorts most likely to benefit and the optimal way to integrate immunotherapy with existing standard-of-care HER2-targeted agents.

## Immune landscape and heterogeneity of HR^+^/HER2^–^ BC

HR^+^ BC represents the vast majority of diagnoses (6, 45), many of them in women under the age of 50 (2, 4). These tumors generally depend on ER signaling for growth and survival (46), though HR^+^ BC is also incredibly common in postmenopausal women (47).

In clinical practice, all HR^+^ tumors are classified under the same umbrella based on IHC testing and are often treated with the same regimens. However, HR^+^ BC subtypes have vastly different biologies and patient outcomes. PAM50 is a 50-gene signature that has been used to categorize breast cancers into five intrinsic subtypes that have distinct clinical outcomes (48). By PAM50 analysis, more than 60% of HR^+^ BCs are classified luminal A, with luminal B cancers making up the second most common subtype (~25%) (45). The minor remaining cases are made up of basal-like, HER2-enriched, and normal-like phenotypes (45, 48). Basal-like and luminal B subtypes are known to be more aggressive than luminal A tumors because of a gene signature that more closely resembles a TNBC-like phenotype, presenting with higher grade or stage (49, 50). Histologically, luminal A tumors can be categorized by high PR expression, low nuclear grade, and low Ki67 (proliferation marker), while luminal B tumors typically have lower PR expression, higher grade, and high Ki67, making them more proliferative and more likely to benefit from chemotherapy (49, 51).

In addition to heterogeneity based on molecular classification, other clinical factors appear to affect disease outcomes and may reflect different underlying disease biology. For example, younger women with HR^+^ BC have worse outcomes than their older counterparts, independent of genomic risk, with some data showing worse outcomes even when matched according to genomic risk scores (52, 53). The TAILORx and RxPONDER studies have also indicated that younger women may derive more benefit from chemotherapy rather than endocrine therapy alone compared with postmenopausal patients (54, 55). This suggests that the benefit from chemotherapy may be driven by early induction of menopause, rendering younger premenopausal women at higher risk of recurrences and mortality.

As noted above, HR^+^ BC has generally been considered nonimmunogenic, mostly based on direct comparisons with the other subtypes (56). However, given the vast heterogeneity of HR^+^ BCs, it is perhaps unsurprising that immune infiltration would also be highly heterogenous. Indeed, a subset of early-stage HR^+^ tumors exhibit a more TNBC-like phenotype, with lower ER expression, correlating with increased TILs (26, 57).

Although the median TILs in HR^+^/HER2^–^ BC is low compared with TNBC, a minority of HR^+^/HER2^–^ tumors have above 30% TILs (26, 58). Higher TIL levels are also associated with lower PR expression in HR^+^ BCs, increased lymph node involvement, and higher tumor grade, correlating to a more luminal B and biologically aggressive phenotype (Figure 1A) (58, 59). This is also true at the transcriptomic level, with expression of *ESR1* negatively correlated with TILs and PD-L1 expression (60). Interestingly, spatial distribution of CD8^+^ TILs has a positive impact on ER^+^ BC patient outcomes (61). Other studies have also highlighted high spatial heterogeneity in the TIL landscape between HR^+^ samples (62), again challenging the notion that HR^+^ BC is a “cold” subtype and encouraging more in-depth characterization of the TME among this subtype.

Homologous recombination deficiency (HRD) has also been linked to the immunogenicity of HR^+^ BCs and their biology (63). HRD refers to the loss of DNA damage proteins, such as *BRCA1/2*, *RAD50/51*, and *PALB2*, or associated genes, such as *ATM* and *CHEK1/2*, through either germline variants or acquired mutations within tumor cells (64–66). Patients harboring mutations in core homologous recombination pathways are highly responsive to DNA repair–targeted treatments, such as poly (ADP-ribose) polymerase (PARP) inhibition (67). HRD tumors have been associated with more inflamed and immunogenic TMEs in breast cancer (68, 69). This may be because HRD is correlated with increased tumor mutation burden (TMB) and thus higher neoantigen load (70). High TMB has been correlated with increased immunogenicity and with APOBEC-driven mutagenic signatures in breast cancers (71). Furthermore, deficiency in *BRCA1/2* has also been correlated with high levels of large genomic rearrangements (72) that may increase the repertoire of potentially immunogenic alterations, including structural variants that are incompletely captured by conventional TMB measures. Neoantigen load and presentation via HLA molecules are essential for T cell engagement, potentially leading to a “hot” immune environment (73). Interestingly, HR^+^/HER2^–^ BCs have very high levels of HLA downregulation (74, 75), an established mechanism of immune escape (76), often as a result of strong immune activation (77). This could indicate that HR^+^ BCs induce a very strong immune reaction in the early stages of development, which then pressures immune escape and ultimately leads to a less inflamed microenvironment.

## Clinical immune modulation in HR^+^/HER2^–^ BC

The prognostic role of TILs in HR^+^/HER2^–^ BCs has been somewhat controversial. While some studies have reported a lack of a correlation between TILs and disease-free survival outcomes in HR^+^ disease (19, 78), the association between higher TILs and more aggressive biology (luminal B, higher grade, and nodal involvement) needs to be considered.

Several early clinical trials evaluating ICB in pretreated HR^+^/HER2^–^ metastatic breast cancer failed to show clinically meaningful response rates, even in PD-L1–positive selected populations (Tables 3 and 4). Nonetheless, some signals for activity were observed, with an ORR of 19% in the ICON study in a cohort who received the anti-CTLA4 antibody ipilimumab in combination with the anti–PD-1 antibody nivolumab sequentially after chemotherapy (79). More recently, efforts have also focused on identifying subgroups more likely to benefit from ICB. The phase II Nimbus study evaluated a combination of ipilimumab and nivolumab in patients with high TMB (TMB > 9 mutations per megabase) in HER2^–^ metastatic breast cancer (both TNBC and HR^+^ BC), with an ORR of 20% (80).

Metastatic disease is associated with a more suppressive immune TME, as well as larger tumor burden. Multiomic analysis of matched primary and metastatic samples in the AURORA studies of relapse in metastatic breast cancer have highlighted the vast heterogeneity of metastatic TMEs, which are heavily context dependent on subtype and site (81, 82). For instance, lymph node or skin metastases were shown to have higher immune scores relative to other metastatic sites, such as the liver (81). Lymph node–only metastases have also been correlated with more durable responses to anti–PD-1 blockade (83), indicating the cellular composition of the metastatic site (particularly the immune cell composition) determines the level of efficacy of ICB. While this constrains our ability to identify broad biomarkers of immunogenicity in metastatic patients, there is also an opportunity to improve our understanding of metastatic TMEs in breast cancer and how best to harness immunity in late-stage disease.

Higher response rates to immunotherapy are seen in early-stage HR^+^ disease, similar to what has been observed in TNBC, particularly when it is administered in the neoadjuvant setting. The GIADA trial was one of the first trials that tested ICB as a neoadjuvant therapy in early-stage HR^+^/HER2^–^ disease (84). The trial showed moderate efficacy in a cohort of 43 luminal B–like early-stage breast cancers, with basal-like phenotypes showing the highest response rates to neoadjuvant nivolumab (84). Subsequently, two large, randomized, phase III studies have evaluated the efficacy of anti–PD-1 antibodies combined with neoadjuvant chemotherapy for HR^+^/HER2^–^ BC: CHECKMATE-7FL (28), evaluating nivolumab, and KEYNOTE-756 (29), evaluating pembrolizumab. Both studies recruited high-grade or lymph node–positive patients with high Ki67, and included patients with low ER expression, to capture this biologically aggressive phenotype. Both studies showed a statistically significant improvement in pCR rate compared with chemotherapy. Most astonishingly, subgroup analysis from CHECKMATE-7FL showed that the addition of nivolumab nearly doubled the pCR rate (24.5% vs. 13.8%) in a BC subtype historically viewed as immune “cold” (28). This is further supported by the PD-L1 subgroup analysis from the KEYNOTE-756 trial showing similarly impressive response rates in this group (29). These two independent studies indicate preexisting immunity that is highly sensitive to the addition of ICB to chemotherapy. The findings are notable because pCR rates in HR^+^ BC have historically remained low with chemotherapy, ADCs, and endocrine therapies. They point to an underappreciated role for T cell biology in shaping prognosis and treatment response and suggest a therapeutic opportunity in a subgroup with historically poor outcomes.

Moreover, the broad variation in TIL within this cohort suggests an opportunity to further sensitize patients to ICB through synergistic combinations. Several studies have explored combinations of ICB with other targeted therapies that appear to (through various mechanisms) contribute to immune priming, including PARP inhibitors, CDK4/6 inhibitors, and ADCs. PARP inhibitors are thought to promote a proimmune microenvironment by activating the cGAS-STING pathway (85), and research has reported synergy with ICB (86). For instance, in the metastatic setting, the MEDIOLA study on HER2^–^ disease showed promising efficacy in combining the PARP inhibitor olaparib with the anti–PD-L1 antibody durvalumab (87). In the early-stage setting, this combination was tested in one arm of the adaptive platform I-SPY2.2 study (88), and further evaluation of this strategy is ongoing in the OLIO trial (ACTRN12623000657628).

Immunomodulatory effects of CDK4/6 inhibitors have also been reported. Although mechanisms remain unclear, upregulation of antigen presentation machinery, downregulation of regulatory T cells, influx of CD8^+^ T cells, and induction of immunological memory have all been observed following treatment with CDK4/6 inhibitors (89–91). Preclinical studies and some limited clinical data have also shown synergy between CDK4/6 inhibitors and ICB (92). However, implementation of this approach has been limited by toxicity and questions of how best to harness the immunomodulatory effects of CDK4/6 inhibitors in the clinical setting.

ADCs are a novel drug class showing efficacy in HR^+^ BC as monotherapy with various targets (93, 94). ADCs with topoisomerase 1 inhibitor (Topo1i) cytotoxic payloads (including T-DXd and Dato-Dxd) have been shown to induce immunogenic cell death (95, 96), and remarkable clinical activity for the combination of Topo1i ADCs and ICB has been observed (97). The I-SPY2.2 trial evaluated this approach in the neoadjuvant setting for patients with early-stage breast cancer, including a cohort of HR^+^/HER2^–^ BC, and showed promising efficacy, particularly in patients with higher immune scores (98).

Overall, it is likely that ICB responses have been modest in past HR^+^/HER2^–^ trials because of design limitations in patient selection. Treatment-related toxicities and the risk of overtreatment of patients who would not benefit from ICB are also key challenges, which must be noted. Multiple biomarkers of immunogenicity, such as TILs and HRD, have been used to highlight immune-active patient populations. However, the integration of these biomarkers into the clinic for the management of HR^+^/HER2^–^ disease has been relatively slow, and will be imperative in improving patient selection (35), as discussed above for TNBC. It is becoming evident that not all HR^+^/HER2^–^ tumors have equivalent TMEs, and there is a clear opportunity for ICB in the clinical management of selected subgroups of HR^+^/HER2^–^ disease, specifically high-risk patients with poorer outcomes under the current clinical management guidelines.

## Mechanisms that regulate immunogenicity in breast cancer

Understanding the mechanisms that govern the balance between immune activation and suppression is critical, particularly in HR^+^/HER2^–^ disease, where these pathways may identify patients most likely to benefit from immune-based therapies. Tumor-intrinsic factors like genomic instability, neoantigen burden, and oncogenic signaling pathways can intersect with tumor-extrinsic influences, including stromal composition, endocrine signaling, and systemic host factors like age and immune fitness. Together these factors are key biological mechanisms shaping tumor immunity and can either enable or restrain immunogenicity in HR^+^/HER2^–^ BC. By dissecting these pathways, we can potentially identify opportunities to enhance immunotherapeutic responsiveness in this historically “cold” subtype.

ER signaling has been implicated in shaping the immune microenvironment of HR^+^/HER2^–^ BC, often promoting immune evasion through modulation of antigen presentation and cytokine networks (99). Immune cells including B, T, and myeloid cells express ERs and can respond directly to estrogen signaling (100). There are two broad classes of ERs, α (*ESR1*) and β (*ESR2*), both of which can be expressed in immune cells (100).

ERα (*ESR1*) response pathways have been shown to suppress antitumor immunity and promote a “colder” immune microenvironment (101). *ESR1* signaling can drive immunosuppressive myeloid programs, which in turn inhibit T cell activation (102, 103). In contrast, blockade of *ESR1* signaling can improve immunogenicity in HR^+^ models of breast cancer and promote T cell recruitment (104) and pro-inflammatory myeloid responses (105). This provides strong evidence for the synergistic combination of hormone blockers and ICB in HR^+^/HER2^–^ BCs. Indeed, emerging results from preclinical models of HR^+^ disease show promising activity with the combination of ICB and hormone-targeted therapy (106).

Interestingly, the opposite seems to be true for ERβ (*ESR2*). While the role of *ESR2* in immune cells remains incompletely defined, recent evidence in murine models suggests that *ESR2* activation in CD8^+^ T cells can enhance T cell receptor signaling and produce a more potent T cell response, which enhances responses to PD-1 blockade (107). However, *ESR2* antagonist monotherapy did not show efficacy in early preclinical testing (108), indicating that *ESR2* signaling alone is unlikely to be sufficient to activate antitumor immunity, though it may have potential for immune priming.

Progesterone has also been implicated in immune regulation. Signals driven by progesterone can act in an immunosuppressive manner systemically, driving antiinflammatory mechanisms in T cells and other cell types (109). In animal models, PR stimulation and overexpression led to increased mammary tumors and reduced intratumoral immunity (110, 111). In line with these data, antiprogesterone treatment can improve T cell fitness (112), which again may have synergistic properties in the context of immunotherapy. Current studies are testing the efficacy of progesterone inhibition and pembrolizumab combination with promising early results (113), though more research is needed to fully understand the interplay of PR signaling and the immune microenvironment.

Endocrine treatment can also influence antigen presentation. Antiestrogen therapy has been shown to increase HLA expression in HR^+^ cancers (114). Similarly, PR signaling can also downregulate HLA expression in mammary tumors (115). The repertoire of antigens presented by HLA appear to be subtype specific, with tumor-specific antigens (including neoantigens) being more prevalent in TNBC compared with HR^+^/HER2^–^ BC (116). To date, common neoantigens are only prognostic in TNBC (116), suggesting that HR^+^/HER2^–^ BCs may have a distinct antigen presentation landscape. For example, *NY-ESO-1* and *WT1* antigens are more highly expressed in TNBC (117), whereas *MUC1* (118) and *PIK3CA* (119) have been associated with increased immunogenicity in HR^+^/HER2^–^ disease. Better characterization of the breast cancer immunopeptidome will be highly beneficial for defining subtype-specific immune vulnerabilities and for developing more precise immunotherapeutic strategies.

In addition, environmental and host factors can affect immunogenicity in HR^+^/HER2^–^ disease. Age, and specifically systemic immune aging, has been correlated with improved outcomes of ICB monotherapy in TNBC (30). Given the importance of age in HR^+^ BC outcome, this factor and its influence on systemic immunity should be considered alongside efficacy data of any new targeting agent that can modulate the immune system. Early studies support this, as high CD8^+^ T cell infiltration was associated with improved survival in HR^+^ disease in premenopausal women, but not postmenopausal women (120). Importantly, endogenous estrogen levels are not the only variable at play. While systemic levels of estrogen decline with age and menopause, aromatase expression within tumors can still lead to an estrogen-rich TME in older patients (121).

Other host factors, such as exercise (122) and nutrition (123), can improve CD8^+^ T cell “fitness” and sensitize tumors to ICB. Parity and lactation duration have also recently been shown to remodel the immune landscape of the normal cancer-unaffected breast and maternal systemic immunity establishing a long-lived population of breast-resident CD8^+^ T cells. (124). In TNBC, reproductive history was associated with increased immune infiltration and favorable outcome, providing a potential mechanistic link between breastfeeding and the established reduction in long-term TNBC risk (124). Importantly, these data provide a biological mechanism for the long-observed association between longer breastfeeding duration and lower risk of TNBC and suggest that an important component of antitumor immunity in breast cancer has been underappreciated. Although the exact mechanisms underlying this immunogenicity remain to be defined, it is plausible these reproductive events also affect immunogenicity of the HR^+^ proliferative breast cancer subtypes, warranting further research. Genetic host factors such as germline variants in *BRCA1* may likewise impact both tumor-intrinsic and systemic immune fitness in carriers (125–127).

Another conceptual shift of importance has come from two recent studies that repositioned the thymus from a pediatric immune organ to an active determinant of immunotherapy efficacy (128, 129), challenging the long-held belief that thymic involution with age renders the organ largely nonfunctional in adults. These findings may have implications for breast cancer, introducing another host-related variable, in this case thymic reserve, that could independently shape the TME.

The association among thymic health, parity history, and T cell receptor clonal diversity raises the possibility that breast cancer immunogenicity may be shaped upstream of the tumor-immune interface. Together, these findings support a model in which host factors are important determinants of immune surveillance and immunotherapy response in breast cancer (Figure 1B), alongside tumor-intrinsic biomarkers, such as TILs, PD-L1, and TMB. Better understanding of how all these factors may drive or restrict immunity, in addition to standardizing ways to quantify them, will be paramount to improve patient stratification in the clinic and avoid the risk of over- or undertreatment.

## Conclusions

Checkpoint inhibitors are now part of standard-of-care treatment for TNBC and provide clear proof of principle that immunotherapy can improve outcomes in breast cancer. Ongoing advances, including a better understanding of host determinants such as parity and thymic reserve, together with the development of new immune-based strategies, including bispecific approaches, are likely to further expand this benefit.

At the same time, the longstanding characterization of HR^+^/HER2^–^ BC as nonimmunogenic is no longer accurate. Emerging evidence clearly demonstrates that immunogenicity exists along a broad spectrum, and there is a subset of HR^+^/HER2^–^, high-grade disease that exhibits substantial immune activity. Better understanding and improved stratification of this immunologically active subgroup are essential for improved patient selection and more effective treatment. Moving forward, the key challenge is no longer determining whether immunotherapy has a role in HR^+^/HER2^–^ disease but in precisely identifying which patients are most likely to benefit from it and how this underappreciated immunogenic potential can be most effectively harnessed.

## Funding support

- SL is a National Breast Cancer Foundation of Australia Endowed Chair.
- SL is supported by the Breast Cancer Research Foundation, New York.

## Figures and Tables

**Figure 1:**
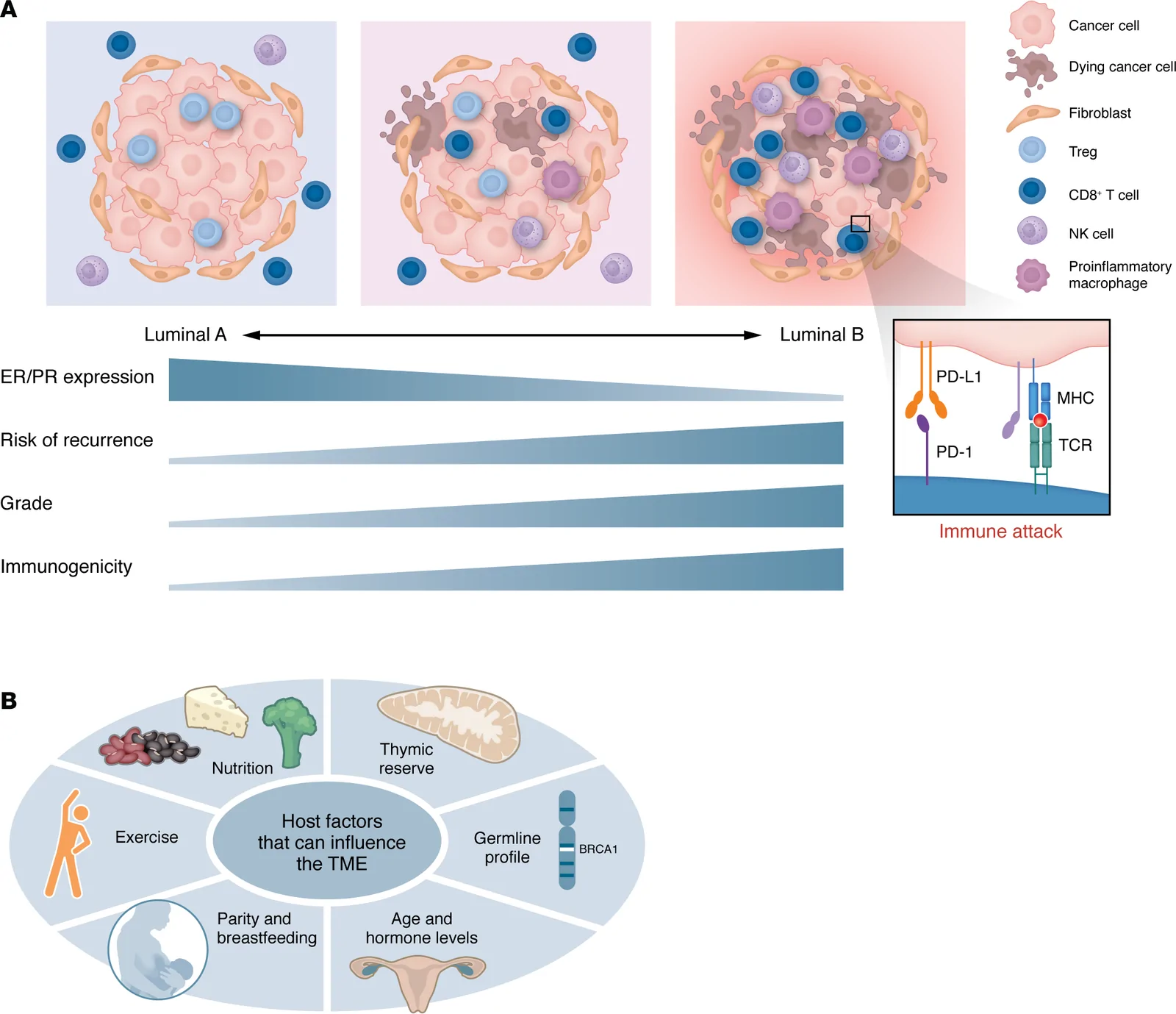
Immunogenicity spectrum within early-stage HR^+^ BC and host factors that may influence the TME. (**A**) The vast heterogeneity of HR^+^ BC leads to a broad spectrum for immunogenicity, which correlates with receptor expression, prognosis, and tumor grade. High-immunogenicity tumors can be more susceptible to ICB. (**B**) Numerous host factors affect tumor immunogenicity.

**Table 4:**
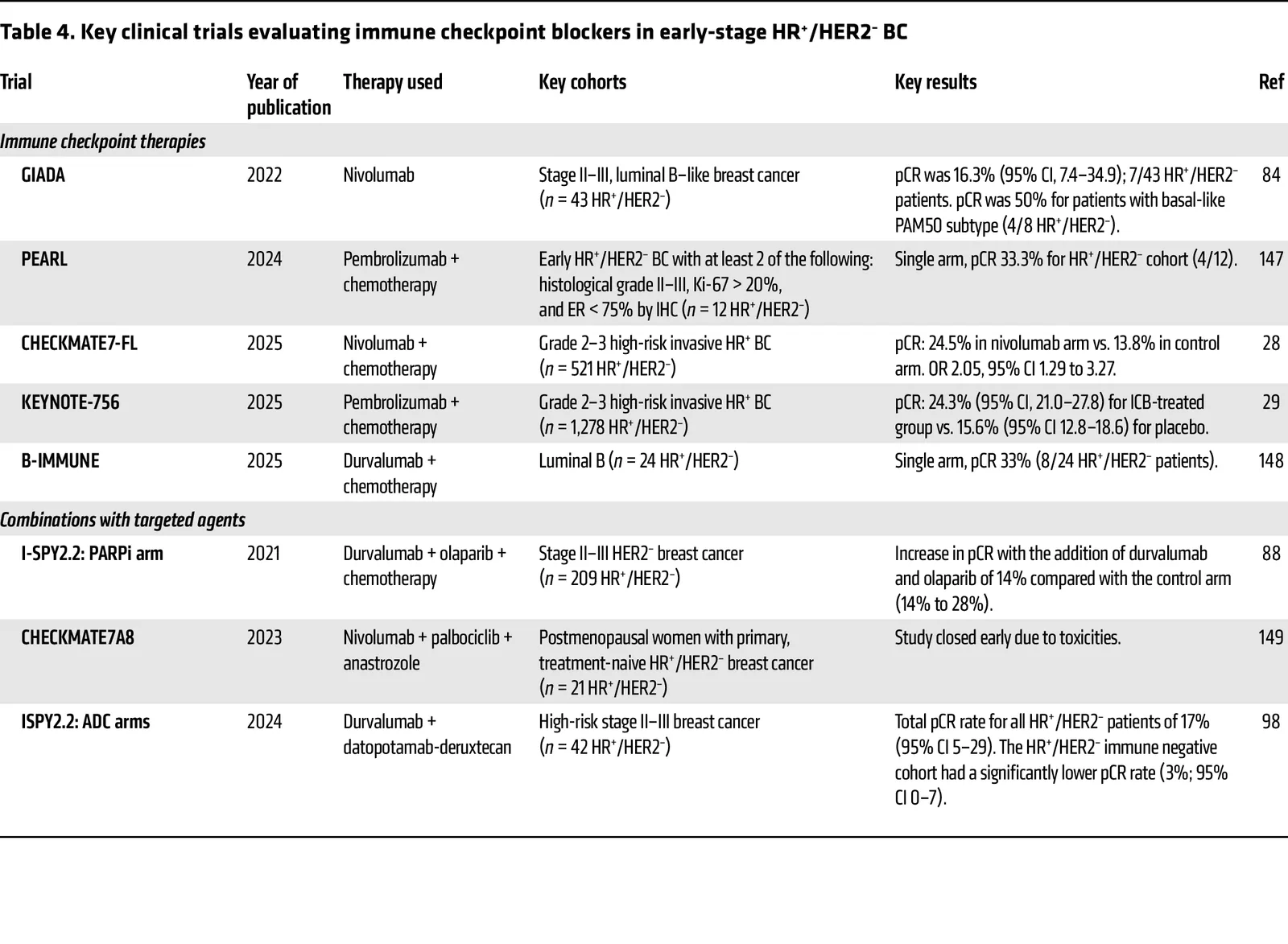
Key clinical trials evaluating immune checkpoint blockers in early-stage HR^+^/HER2^–^ BC

**Table 3:**
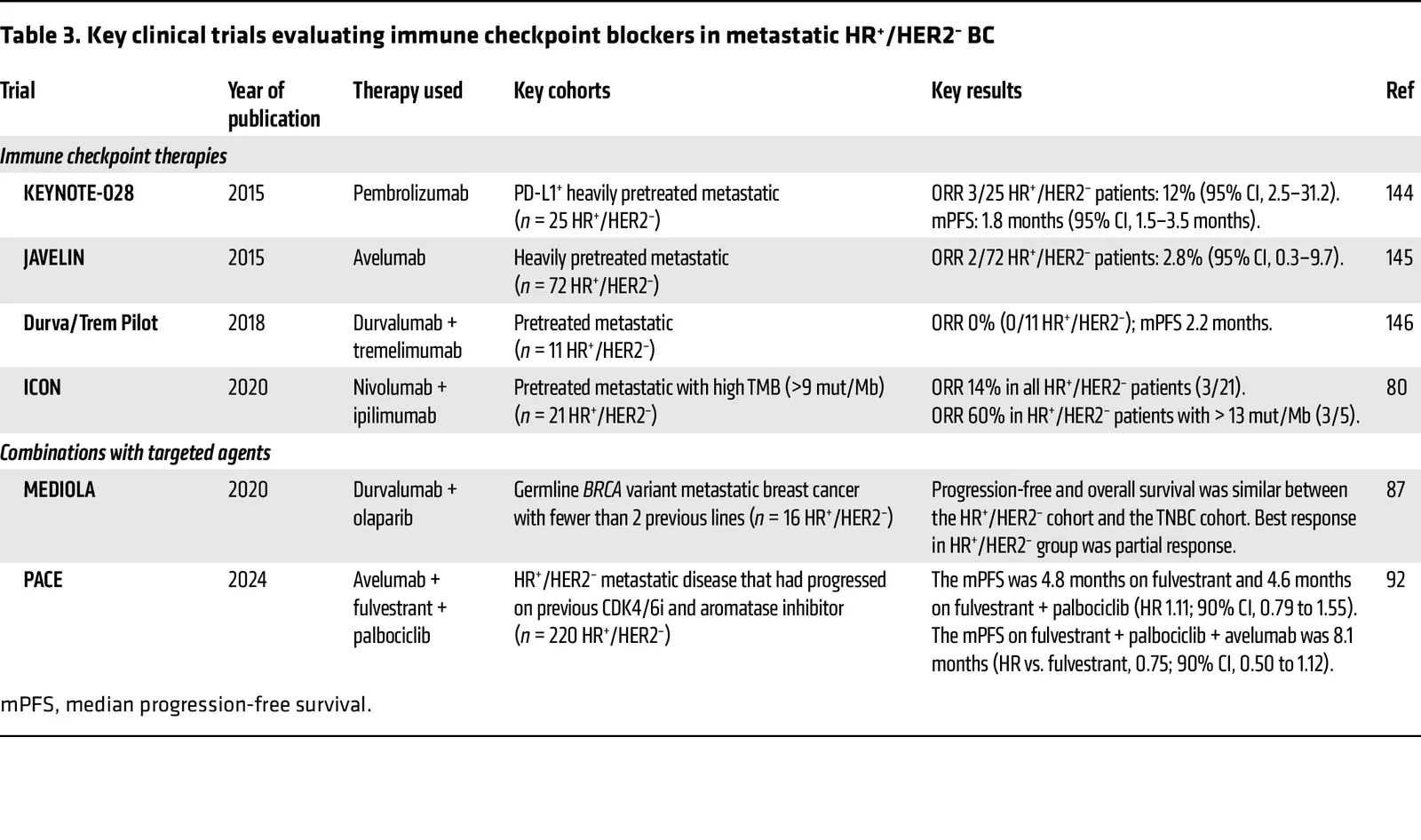
Key clinical trials evaluating immune checkpoint blockers in metastatic HR^+^/HER2^–^ BC

**Table 2:**
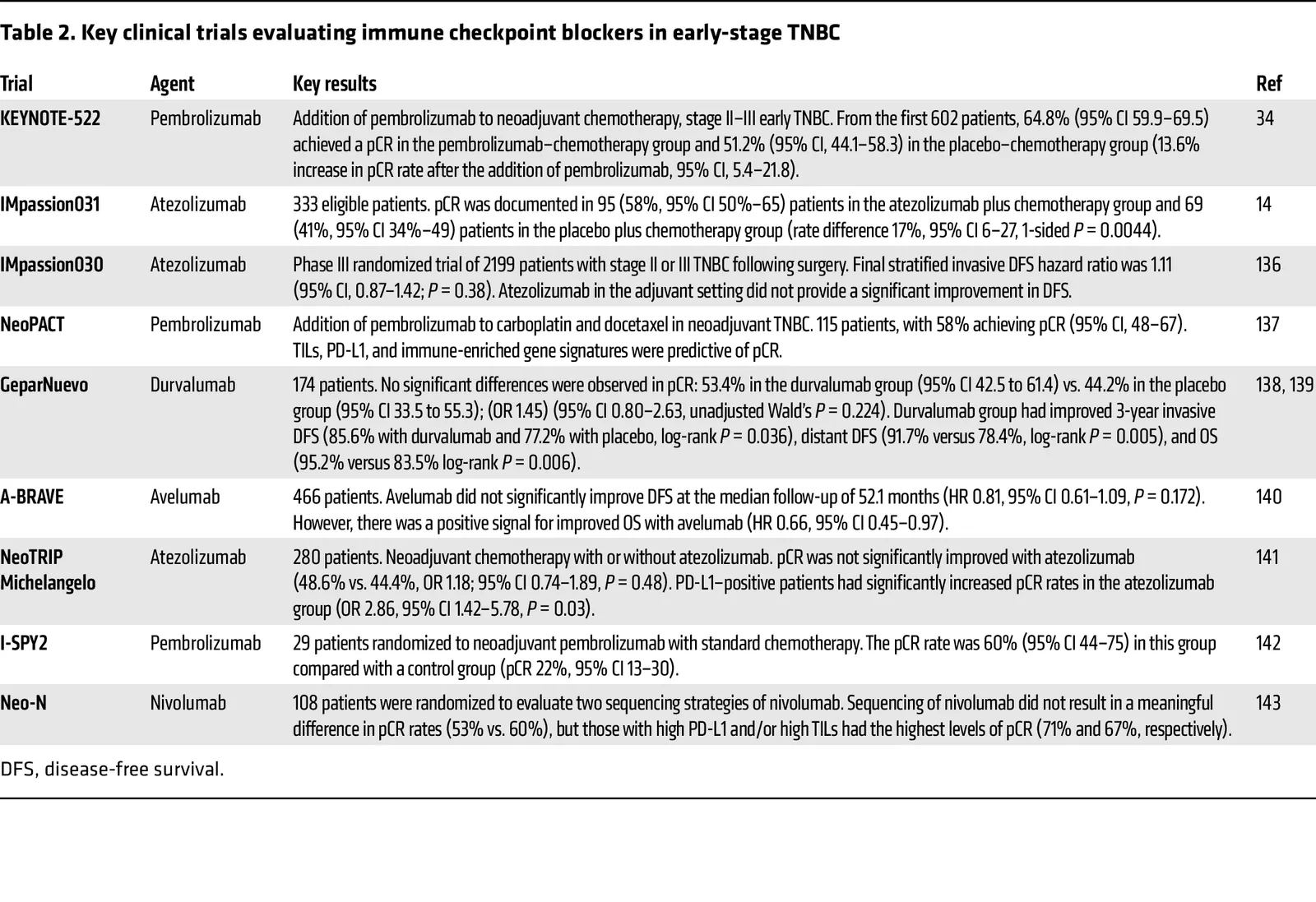
Key clinical trials evaluating immune checkpoint blockers in early-stage TNBC

**Table 1:**
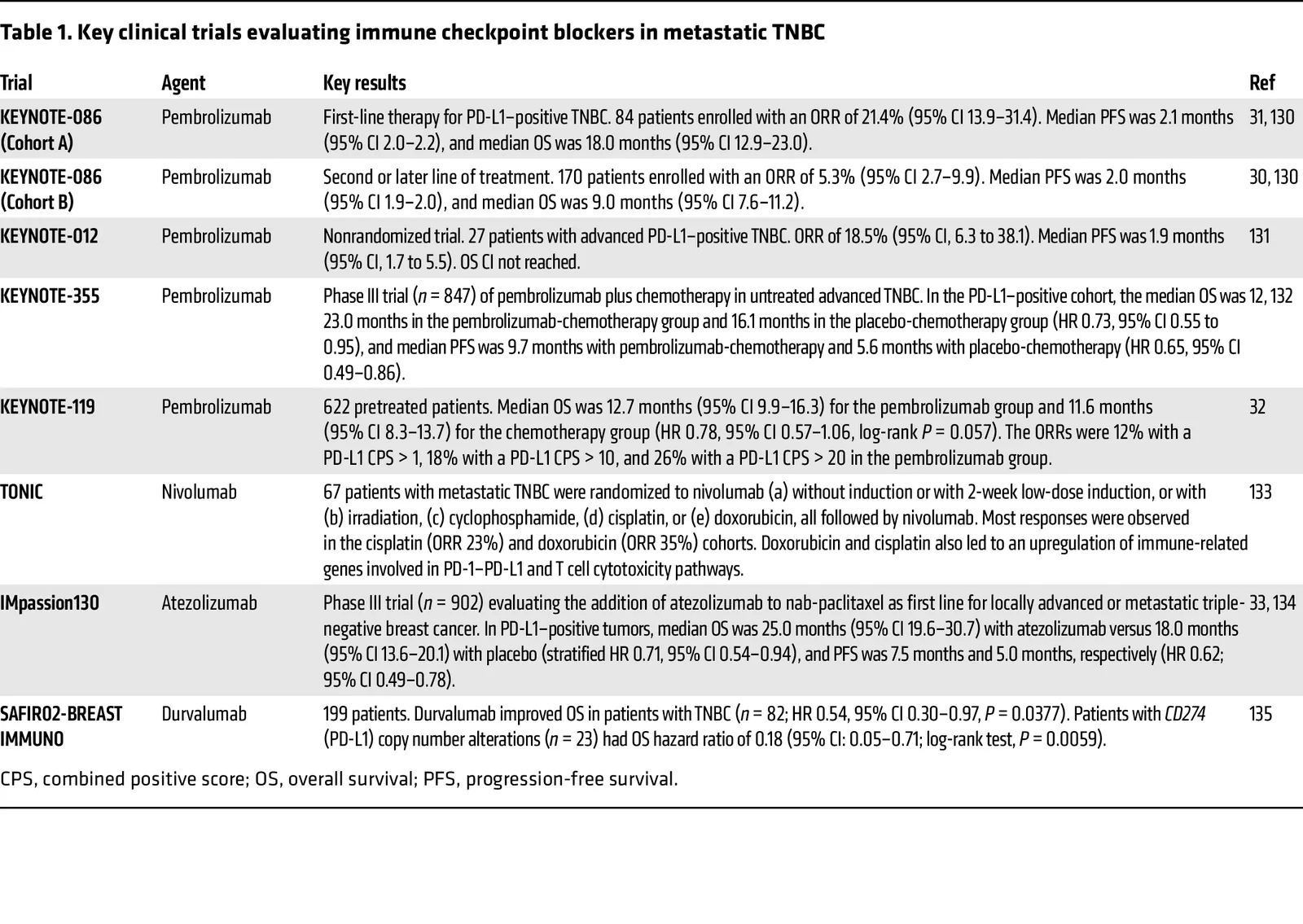
Key clinical trials evaluating immune checkpoint blockers in metastatic TNBC

## Version 1 10/01/2026

Electronic publication
